# Effects of supplemental methionine sources in finishing pig diets on growth performance, carcass characteristics, cutting yields, and meat quality

**DOI:** 10.1093/tas/txae088

**Published:** 2024-05-27

**Authors:** Hannah M Remole, John K Htoo, S M Mendoza, Casey L Bradley, Ryan N Dilger, Anna C Dilger, Bailey N Harsh

**Affiliations:** Department of Animal Sciences, University of Illinois, Urbana, IL, USA; Evonik Operations GmbH, Hanau-Wolfgang, Germany; Evonik Corporation, Kennesaw, GA, USA; The Sunswine Group, Lowell, AR, USA; Department of Animal Sciences, University of Illinois, Urbana, IL, USA; Department of Animal Sciences, University of Illinois, Urbana, IL, USA; Department of Animal Sciences, University of Illinois, Urbana, IL, USA

**Keywords:** carcass composition, meat quality, methionine, pork, sulfur amino acid, swine nutrition

## Abstract

Supplemental methionine (Met) is widely used within the swine industry; however, data are limited regarding the effect of Met sources on carcass cutability and meat quality. The objective was to determine the effects of L-Met (LM, 99%), DL-Met (DLM, 99%), or calcium salt of DL-Met hydroxyl analog (MHA, 84%) in finishing pig diets on carcass characteristics and meat quality. At 9 weeks of age, pigs (*N* = 240) were allocated to 60 single-sex pens for a four-phase finishing trial that lasted 104 d. Pigs were fed a common grower diet until day 56 where pens were randomly allotted to one of the three experimental diets. For the remaining 7 wk of the finisher phase, pigs (BW = 79.9 ± 0.80 kg) were fed diets containing LM, DLM, or MHA, with the supplemental Met source providing 25% of standardized ileal digestible (SID) Met + cysteine (Cys) requirement based on 65% bioefficacy for MHA in comparison with LM or DLM. One pig per pen was slaughtered at the study conclusion (on day 104), and the left sides of carcasses were fabricated into subprimal cuts to determine carcass-cutting yields. Loin quality including proximate composition and shear force were measured. Hot carcass weight was not different (*P *= 0.34) between treatments (LM 104.5 kg; DLM 103.0 kg; MHA 101.5 kg), moreover, loin eye area was not different (*P *= 0.98) between treatments (LM 52.65 cm²; DLM 52.49 cm²; MHA 52.81 cm²). Boneless carcass-cutting yield was not different (*P *= 0.56) between treatments (LM 54.97 kg; DLM 54.82 kg; MHA 54.52 kg). Loin pH was not different (*P *= 0.24) between treatments (LM 5.45; DLM 5.48; MHA 5.45). However, drip loss tended to be reduced (*P *= 0.11) by the DLM treatment (5.58%) compared with LM (7.03%) and MHA (6.68%) treatments. Shear force was not different (*P *= 0.85) between treatments (LM 3.03 kg; DLM 3.06 kg; MHA 3.10 kg). However, cook loss tended to be reduced (*P *= 0.06) by the DLM treatment (16.20%) compared with LM (18.18%) and MHA (18.50%) treatments. These data suggest that only minimal differences in carcass cutability and meat quality can be attributed to Met source in finishing pig diets when using 65% bioefficacy for MHA relative to L-Met or DL-Met.

## Teaser

Supplemental methionine source (L-Met, DL-Met, or MHA) can be fed in finishing pig diets with little impact on growth performance, cutting yields, fresh loin, and belly quality when using 65% bioefficacy for MHA relative to L-Met or DL-Met. This lack of differences indicates there would be little advantage or disadvantage for carcass yield and quality to using one Met source over another.

## Introduction

Methionine (Met) is an essential amino acid (AA) with important roles in the synthesis of S-adenosyL-Methionine (SAM), methylation, protein synthesis, and is involved in anti-oxidation biological processes (Levine et al., 1996). Methionine supplementation is common in commercial growing-finishing pig diets because the high Met requirements of pigs coupled with low Met concentrations in industry-typical corn–soybean diets make Met the second limiting AA for pigs (Fontecave et al., 2004; Courtney-Martin & Pencharz, 2016).

Historically, supplementary Met has been commonly supplied as: DL-Met (99% pure) or a DL-methionine hydroxyl analog (MHA). MHA can be produced in liquid or solid form as a free acid (MHA-FA, 88%) or calcium salt (MHA-Ca, 84%). Until recently, L-Met has not been used in commercial production due to the high cost of purification. However, novel microbial fermentation processes have recently led to some commercial availability of feed-grade L-Met (99% pure). DL-Met is a 50:50 mixture of the D and L enantiomers, while MHA (2-hydroxy-4-methylthio butanoic acid) is a 50:50 mixture of L-HMTBA and D-HMTBA isomers but is chemically different from DL-Met due to the replacement of the amino group by a hydroxyl group (Yang et al., 2020). To be utilized for protein synthesis in the pig, all D-Met or DL-MHA must be converted to L-Met before their effective utilization by cells, whereas only the D-isomer in DL-Met needs to be converted to L-Met (Dilger & Baker, 2008). Calcium salt of MHA contains water (2%) and calcium (14%) and is commonly assumed to have an average bioefficacy of 65% on a product basis in comparison with DL-Met (Opapeju et al., 2012; Wang et al., 2020). Despite these differences, it is largely accepted that when formulated for the same bioavailable Met content, few differences in pig growth performance are observed (Zimmerman et al 2005; Opapeju et al 2012).

Although previous research suggests no differences in growth performance between the liquid or calcium salt of MHA compared with DL-Met when using 65% bioefficacy for MHA (Zimmermann et al. 2005; Opapeju et al., 2012; Wang et al., 2020), a recent report has indicated differences in loin chop lightness (L*) and pH at 45 min postmortem between carcasses from pigs supplemented with liquid MHA or DL-Met (Yuan et al., 2023). However, few other reports exist of differences in meat quality or lean yield between Met sources. Therefore, the objective of this study was to determine the effects of three Met sources on growth performance, pork carcass characteristics, and meat quality. Overall, it was hypothesized that feeding different Met sources would have little impact on lean yield or quality when supplemented by considering differences in their relative bioefficacy.

## Materials and Methods

All animal care and use procedures were approved by the Institutional Animal Care and Use Committee at the University of Illinois (Protocol #23,045) and followed standard practices described in the Guide for the Care and Use of Agricultural Animals in Research and Teaching (2020).

### Dietary Treatments

Pigs were fed ad libitum for 104 d using a four-phase feeding system. From day 0 (9 wk of age) to day 55, pigs were fed common, phased grower diets (Supplementary Table 1) formulated to meet the nutritional requirements of pigs 25 to 50 kg (Grower 1) and 50 to 75 kg (Grower 2), respectively (Swine NRC, 2012). From days 56 to 104, pigs were fed assigned experimental diets. The experimental diets for phase 3 (days 56 to 83) and phase 4 (days 84 to 104) were designed to meet 75% of the estimated standardized ileal digestible (SID) Met + Cys requirements of pigs weighing 75 to 100 kg and 100 to 135 kg, as shown in Table 1 (Swine NRC, 2012). One of the three supplemental Met sources (L-Met [LM], DL-Met [DLM], or MHA [MHA]) was then added to the low-CP diets to satisfy the estimated Met requirement (Table 1). Bioefficacy of L-Met and DL-Met was assumed to be 100% in accordance with Swine NRC guidelines (2012), whereas that of MHA was assumed to be 65% based on previous studies (Opapeju et al., 2012; Wang et al., 2020).

**Table 1.** Calculated ingredient and nutrient compositions of experimental diets (as fed basis)

	Phase 3 (days 56 to 83)			Phase 4 (days 84 to 104)		
Item	L-Met	DL-Met	MHA	DL-Met	L-Met	MHA
*Ingredient, %*						
Corn	33.71	33.71	33.71	19.43	19.43	19.43
Sorghum	35.00	35.00	35.00	60.00	60.00	60.00
Soybean meal	2.50	2.50	2.50	.	.	.
Field peas	25.00	25.00	25.00	17.00	17.00	17.00
Soybean oil	1.00	1.00	1.00	1.00	1.00	1.00
Limestone	0.84	0.84	0.84	0.83	0.83	0.83
Dicalcium phosphate	0.55	0.55	0.55	0.35	0.35	0.35
L-Lys.HCl	0.26	0.26	0.26	0.32	0.32	0.32
L-Thr	0.12	0.12	0.12	0.09	0.09	0.09
L-Trp	0.05	0.05	0.05	0.03	0.03	0.03
L-Val	0.02	0.02	0.02	0.00	0.00	0.00
DL-Met	0.00	0.11	0.00	0.09	0.00	0.00
L-Met	0.11	0.00	0.00	0.00	0.09	0.00
MHA^*^	0.00	0.00	0.17	0.00	0.00	0.14
Cornstarch	0.29	0.29	0.23	0.31	0.31	0.26
Salt	0.40	0.40	0.40	0.40	0.40	0.40
Vitamin-mineral premix^†^	0.15	0.15	0.15	0.15	0.15	0.15
*Calculated composition*						
ME, kcal/kg	3,397	3,397	3,394	3,445	3,445	3,443
NE, kcal/kg	2,532	2,532	2,530	2,623	2,623	2,621
Ca, %	0.52	0.52	0.52	0.46	0.46	0.46
P^‡^, %	0.24	0.24	0.24	0.21	0.21	0.21
Crude protein, %	12.79	12.79	12.79	10.99	10.99	10.99
*SID AA* ^‖^ *, %*						
Arg	0.72	0.72	0.72	0.53	0.53	0.53
His	0.27	0.27	0.27	0.23	0.23	0.23
Ile	0.41	0.41	0.41	0.34	0.34	0.34
Leu	1.03	1.03	1.03	0.98	0.98	0.98
Lys	0.73	0.73	0.73	0.61	0.61	0.61
Met	0.27	0.27	0.27	0.24	0.24	0.24
Met + Cys	0.42	0.42	0.42	0.36	0.36	0.36
Phe	0.51	0.51	0.51	0.44	0.44	0.44
Thr	0.48	0.48	0.48	0.40	0.40	0.40
Trp	0.15	0.15	0.15	0.12	0.12	0.12
Val	0.50	0.50	0.50	0.41	0.41	0.41

^*^Abbreviations: MHA = Calcium salt of Met hydroxyl analog and supplemented based on 65% bioefficacy for MHA relative to DLM.

^†^The vitamin-mineral premix will provide the following quantities of vitamins and micro-minerals per kilogram of complete diet: Vitamin A as retinyl acetate, 11,150 IU; vitamin D3 as cholecalciferol, 2,210 IU; vitamin E as DL-alpha-tocopheryl acetate, 66 IU; vitamin K as menadione nicotinamide bisulfate, 1.42 mg; thiamin as thiamin mononitrate, 1.10 mg; riboflavin, 6.59 mg; pyridoxine as pyridoxine hydrochloride, 1.00 mg; vitamin B12, 0.03 mg; D-pantothenic acid as -calcium pantothenate, 23.6 mg; niacin, 44.1 mg; folic acid, 1.59 mg; biotin, 0.44 mg; Cu, 20 mg as copper chloride; Fe, 125 mg as iron sulfate; I, 1.26 mg as ethylenediamine dihydriodide; Mn, 60.2 mg as manganese hydroxychloride; Se, 0.30 mg as sodium selenite and selenium yeast; and Zn, 125.1 mg as zinc hydroxychloride.

^‡^Standardized total tract digestible P.

^‖^Amino acids are indicated as standardized ileal digestible AA based on AMINODat 5.0 Platinum version, 2016. (Evonik Nutrition & Care GmbH, Hanau-Wolfgang, Germany).

### Experimental Design

A total of 240 pigs (PIC 357 × Camborough sows) were raised in three separate blocks. The experimental design was a 2 × 3 factorial arrangement of sex and Met source with 10 pens per treatment combination. Prior to trial initiation (day 0), pigs were allotted to treatments by sex and body weight (BW) to minimize variation between pens for starting weight. Pigs were housed in single-sex pens with four pigs per pen (1.18 m^2^/pig) where Met source was randomly assigned to pen (days 56 to 104). A total of 60 pens were used, each containing a nipple waterer, single-space dry-box feeder, and tri-bar slatted floors. Average initial BW was 24.38, 23.51, and 29.14 kg for blocks 1 (*n* = 27 pens), 2 (*n* = 18 pens), and 3 (*n* = 15 pens), respectively.

### Diet Analyses

Analyzed nutrient composition of diets for phases 3 and 4 are presented in Table 2. Dry matter content in the diets was measured by drying in an oven at 103 °C for 4 h. Dietary crude protein content was determined as N × 6.25 using Leco FP-2,000 (Leco Corp., St. Joseph, MI) analyzer. Diets were analyzed for total AA using ion-exchange chromatography with postcolumn derivatization with ninhydrin. Amino acids were oxidized with performic acid, which was neutralized with Na metabisulfite (Llames and Fontaine, 1994). Amino acids were liberated from the protein by hydrolysis with 6 *N* hydrochloric acid for 24 h at 110 °C and quantified with the internal standard by measuring the absorption of reaction products with ninhydrin at 570 nm. Tryptophan was determined by high-performance liquid chromatography with fluorescence detection (extinction 280 nm, emission 356 nm), after alkaline hydrolysis with barium hydroxide octahydrate for 20 h at 110 °C (Commission Directive, 2000). The concentrations of supplemented Met were determined by extraction with 0.1 *N* HCL (European Community, 1998). The concentration of MHA-Ca was determined according to the method described by VDLUFA (1997).

**Table 2.** Analyzed nutrient composition of diets for Phases 3 and 4 (%, as fed basis)

	Phase 3 (days 56 to 83)			Phase 4 (days 84 to 104)		
Analyzed values	L-Met	DL-Met	MHA	DL-Met	L-Met	MHA
Dry matter	87.78	87.60	87.73	87.30	87.28	87.38
Crude protein	16.54	16.20	17.03	12.68	12.55	12.61
Met, total	0.32	0.33	0.23	0.26	0.28	0.19
Met, supplemental	0.09	0.11	<0.01	0.08	0.10	<0.01
MHA	<0.01	<0.01	0.16	<0.01	<0.01	0.13
Cys	0.25	0.24	0.25	0.20	0.20	0.21
Met + Cys	0.57	0.57	0.48	0.45	0.48	0.39
Arg	1.09	1.06	1.10	0.72	0.75	0.75
Ile	0.69	0.66	0.68	0.50	0.49	0.52
His	0.41	0.39	0.41	0.30	0.29	0.30
Leu	1.42	1.35	1.41	1.17	1.16	1.24
Lys	0.91	0.89	0.93	0.67	0.70	0.71
Phe	0.81	0.78	0.82	0.60	0.60	0.63
Trp	0.20	0.19	0.20	0.15	0.14	0.15
Thr	0.67	0.65	0.70	0.51	0.50	0.51
Val	0.78	0.75	0.78	0.59	0.58	0.60
Gly	0.68	0.66	0.69	0.49	0.49	0.50
Ser	0.78	0.76	0.80	0.58	0.58	0.60
Pro	0.90	0.86	0.89	0.75	0.73	0.77
Ala	0.88	0.84	0.88	0.76	0.76	0.80
Asp	1.65	1.60	1.67	1.12	1.14	1.16
Glu	2.91	2.81	2.95	2.22	2.20	2.32

Abbreviations: MHA, calcium salt of met hydroxyl analogue.

### Growth Performance

During the 104-d feeding trial, pigs were individually weighed using a Digi-Star EZ400 scale at the start of each diet phase and at the end of the trial; phase 1 (days 0 to 27), phase 2 (days 27 to 56), phase 3 (days 56 to 83), and phase 4 (days 83 to 104). Feed consumption of pens was recorded on the same days as pig weights were collected. Data collected during the growth period was used to calculate average daily feed intake (ADFI), average daily gain (ADG), and gain:feed ratio (G:F).

### Blood Urea Nitrogen

Pigs were weighed on day 55 to determine the pig closest to the pen average, and this same pig was used for blood urea nitrogen (BUN), carcass quality, and yield evaluations. At days 55, 83, and 101, whole blood was collected via jugular venipuncture using 20-gauge, 2.54-cm vacutainer needles (EDTA BD Vacutainers; Thermo Fisher Scientific, Frederick, MD). Pigs were fasted for 4 to 6 h prior to blood collection. Samples were placed on ice immediately after collection and centrifuged at 1,300 × *g* at 4 °C for 15 min. Plasma fraction was separated and aliquoted into cryovials before storing at −20 °C pending analysis.

For determination of BUN, plasma samples were allowed to thaw at ambient temperature (approximately 21 °C), and subsequently diluted with deionized water at 1:20 (plasma:water) as per manufacturer’s instructions (Urea Nitrogen Colorimetric Detection Kit; Thermo Fisher Scientific, Frederick, MD). An internal standard curve (10, 5, 2.5, 1.25, 0.625, 0.3125, 0.156, and 0 mg/dL) was made using a urea nitrogen stock solution at 100 mg/dL. BUN was measured in 96-well plates with light absorbance measured at 450 nm using a Synergy HT microplate reader (BioTek, Winooski, VT). Concentrations of BUN were calculated using a standard curve and reported as the average of duplicate samples. Intra- and inter-assay precision of BUN kit protocol specified coefficient of variations were calculated by the manufacturer as 2.8 and 4.3, respectively.

### Slaughter and Carcass Characteristics

On day 104, one pig per pen (pig closest to pen average at day 55) was transported to the University of Illinois Meat Science Lab and held overnight in lairage without feed but with ad libitum access to water. On day 105, ending live weight was recorded immediately before slaughter using a GSE Model 350 scale (GSE Scale Systems, Novi, MI). Pigs were immobilized using head-to-heart electrical stunning (Best & Donovan Hog Stunner Model ES, Best & Donovan, Cincinnati, OH) and terminated via exsanguination under the supervision of the Food and Safety Inspection Service of the United States Department of Agriculture. Hot carcass weight (HCW) was recorded approximately 45 min after exsanguination using a Toledo Scale 8,136 rail scale (Toledo Scale, Columbus, OH). Measurement of 45-min longissimus thoracis and lumborum (LTL) muscle pH was taken on left sides, probe was inserted between the 10th and 11th rib in the LTL. Forty-five minute pH was measured using a Hanna Foodcare Portable pH Meter (Hanna Instruments, Woonsocket, RI) calibrated at 4 °C with a Hanna glass electrode (Hanna 4,198,163 pH meter, −2.0 to 20.0 pH/±2000.0 mV; Hanna FC2323 meat specific electrode; 2-point calibration; pH 4 and pH 7).

Carcass sides were allowed to chill in chilling cooler (60,000 BTU, three 40-min defrost cycles/d; Copeland air-cooled refrigeration unit, Copeland LLC, Saint Louis, MO) for at least 20 h at 4 °C. The left side of each carcass was then separated between the 10th and 11th rib to expose the LTL. The anterior face of the exposed LTL was traced on acetate paper. This was later traced using a Wacom digital tracing pad (Wacom, Vancouver, WA) and Adobe Photoshop CS6 (Adobe Inc., San Jose, CA, USA) was used to determine the area. This process was repeated and the average of the two measurements were recorded as loin muscle area. Back fat thickness was measured at ¾ the distance of the LTL from the dorsal process of the vertebral column. Fat-free lean (**FFL**) was calculated using the following equation described by Burson and Berg et al. (2001):


$$\begin{array}{l} \begin{array}{lllll} & Standardized\;fat-free\;lean\;\%= & (8.588+(0.465\times HCW,lb)\; & -(21.896\times fat\;depth,\;in)\; & +(3.005\times LTL\;area,\;in^{2})÷HCW)\times 100 \end{array} \end{array}$$

### Carcass Fabrication

At approximately 22 h postmortem, carcasses were fabricated into primal and subprimal cuts to calculate cutting yields according to the methods described by Boler et al. (2011). The left side of each chilled carcass was weighed using a GSE model 455 rail scale (GSE Scale Systems, Novi, MI) and fabricated into a pork leg (NAMP #401), skin-on whole loin, pork shoulder (NAMP #403), neck bones (NAMP #421), jowl (NAMP #419), skin-on natural fall belly (NAMP #408), and spareribs (NAMP #416) to meet the specifications described in the North American Meat Buyer’s Guide (NAMI, 2014). Each primal was weighed using a Mettler Toledo Model ICS439 scale (Mettler Toledo, Columbus, OH) before further fabrication. Skinned and trimmed hams were further fabricated into a five-piece ham using the Boler et al. (2011) method. The loins were separated into anterior and posterior portions between the 10th and 11th rib and were skinned and trimmed to the specifications of NAMP #410 bone-in loin. Both portions were weighed to determine the weight of the whole skinless bone-in loin. Each portion was then fabricated into a NAMP #414 Canadian back loin, NAMP # 415A tenderloin, and NAMP #413D sirloin. Whole shoulders were skinned and trimmed to meet the specifications of a NAMP #404 skinned pork shoulder. Skinned shoulders were further fabricated into bone-in Boston butt (NAMP #406) and bone-in picnic shoulder (NAMP #405). Bones were removed to produce a boneless Boston butt (NAMP #406A) and a boneless picnic (NAMP #405A) with triceps brachii. Canadian back loins and natural fall bellies were reserved for later evaluation. Carcass-cutting yields were determined using the following equations:


$$\begin{array}{l} Bone-in\;lean\;cutting\;yield,\;\%=\left[\begin{array}{lllll} & (trimmed\;ham(NAMP\;\#402),\;kg\; & +bone-in\;trimmed\;Boston\;butt\;(NAMP\;\#406),\;kg\; & +bone-in\;picnic(NAMP\#405),\;kg\; & +trimmed\;loin(NAMP\;\#410),\;kg)÷chilled\;left\;side\;weight,\;kg\; \end{array}\right]\times 100 \end{array}$$

$$\begin{array}{l} Boneless\;carcass\;cutting\;yield,\;\%=\left[\begin{array}{llllllllll} & \left(\begin{array}{llll} & inside\;ham\left(NAMP\;\;\#\;402F\right),\;kg\; & +outside\;ham\left(NAMP\;\;\#\;402E\right),kg\; & +knuckle\left(NAMP\;\;\#\;402H\right),kg\; \end{array}\right)\; & +inner\;shank,\;kg+lite\;butt,kg\; & +Canadian\;back(NAMP\;\#414),kg\; & +tenderloin(NAMP\;\#415A),kg\; & +sirloin(NAMP\;\#413D),kg)\; & +boneless\;Boston\;butt(NAMP\;\#406A),kg\; & +boneless\;picnic(NAMP\;\#405A),kg\; & +natural\;fall\;belly(NAMP\;\#408),kg)\; & +spareribs\left(NAMP\;\#416\right))\;÷\;chilled\;left\;side\;weight\; \end{array}\right]\times 100 \end{array}$$

$$\begin{array}{l} Boneless\;carcass\;cutting\;yield,\;\%\;=\left[\begin{array}{lllllll} & \left(\begin{array}{lll} & inside\;ham\left(NAMP\;\;\#\;402F\right),kg+outside\;ham\left(NAMP\;\;\#\;402E\right),\;\; & kg+knuckle\left(NAMP\;\;\#\;402H\right),kg\; \end{array}\right)\; & +inner\;shank,kg+lite\;butt,kg+Canadian\;back\left(NAMP\;\;\#\;414\right),kg\; & +tenderloin\left(NAMP\;\;\#\;415A\right),kg+sirloin\left(NAMP\;\;\#\;413D\right),kg)\; & +boneless\;Boston\;butt\left(NAMP\;\;\#\;406A\right),kg+boneless\;picnic\left(NAMP\;\;\#\;405A\right),kg\; & +natural\;fall\;belly\left(NAMP\;\;\#\;408\right),kg)+spareribs\left(NAMP\;\;\#\;416\right))\; & \;÷\;chilled\;left\;side\;weight\; \end{array}\right]\;\times \;100 \end{array}$$

### Loin Quality

At 1 d postmortem, loins were re-faced at the LTL surface posterior to the 10th rib. The portion removed while re-facing was used for the evaluation of drip loss through the suspension method outlined by Boler et al. (2011).

Loin quality traits were evaluated on the ventral loin surface and re-surfaced, anterior chop face of boneless Canadian back loin at the approximate location of the 10th rib. Loins were allowed to bloom for at least 20 minutes on both the ventral surface and chop face before color measurements were collected. Ultimate pH was measured using the same probe as 45 min pH. Instrumental CIE L* (lightness), a* (redness), and b* (yellowness) measurements (Commission Internationale de l’Eclairage (CIE), 1976) were measured using a Minolta CR-400 Chroma meter (Konica Minolta, Osaka, Japan) with a 2° observer, an 8 mm closed aperture, a D65 illuminant, and calibrated with a machine-specific white tile. Subjective NPPC color (NPPC, 1999), marbling (NPPC, 1999), and firmness (NPPC, 1991) were evaluated by a trained technician on the bloomed ventral loin surface and chop face of each loin. Loins were cut into 2.54 cm thick chops posterior to the 10th rib. The first two chops were vacuum packaged in nylon EVA vacuum pouches (O_2_ transmission rate = 57.97 mL·m^2^ ·d-1, 0.076 mm gauge [3 mil]; Charter NEX Films, Inc., Chicago, IL) and stored for proximate analysis and Warner Bratzler Shear Force (WBSF). Proximate analysis chops were trimmed of external fat and frozen at −20 °C prior to analysis. Shear force chops were vacuum-packaged and aged for 14 d at 4 °C before being stored at −20 °C pending analysis.

### Belly Quality

Procedures outlined by Kyle et al. (2014) were used to evaluate fresh belly characteristics. Length was measured at the anterior to posterior midline, and width was measured at the dorsal to ventral midline of the belly. Belly thickness was reported as the average of eight individual locations on the belly and were evaluated by inserting a probe through the lean side of each belly. Measurements 1 to 4 were collected at the midpoint between the latitudinal axis and the dorsal edge at 20%, 40%, 60%, and 80% of the length of the belly, respectively, starting at the anterior end. Measurements 5 to 8 were collected at the midpoint between the longitudinal axis and the ventral edge at 20%, 40%, 60%, and 80% of the length of the belly, respectively, starting at the anterior end. Belly flop was determined by placing the bellies skin side down over a metal bar and measuring the distance between the inside edges of each end.

### Loin Proximate Composition

Moisture and fat percentages of loin chops were determined using the chloroform:methanol extraction procedures described by Novakofski et al. (1989). In brief, chops were allowed to thaw for at least 60 min at approximately 23 °C and then homogenized with a food processor (Hamilton Beach, model 70,720, Glen Allen, VA). Duplicate 10 g samples were dried in a convection oven set at 110 °C for a minimum of 24 h and then weighed. Fat was then extracted using the soxhlet method where the sample was washed in a 4:1 chloroform:methanol mixture for a minimum of 8 h. Samples were dried again in a convection oven set at 110 °C for a minimum of 24 h and weighed. Weights after drying and lipid extraction were used to calculate reported moisture and fat percentages.

### Cook Loss and Warner-Bratzler Shear Force

Chops used for cook loss and WBSF were thawed at 4 °C for a minimum of 24 h and weighed using a Mettler Toledo Model MS802S scale (Mettler Toledo, Columbus, OH) before cooking on Farberware Open Hearth grills (model 455N, Walter Kidde, Bronx, NY, USA). Temperature was continuously monitored during cooking using thermocouples (type K, range: −200 °C to 1,250 °C, standard error: ± 2.2 °C, Omega Engineering, Stamford, CT, USA) placed in the geometric center of chops. Thermocouples were connected to Omega HH378 Data Logger Thermometer (Omega Engineering, Norwalk, CT). Chops were cooked on one side until they reached 31 °C, then flipped and cooked on the other side until reaching 63 °C, at which point they were removed from grills. Chops were cooled to room temperature and were weighed a second time to calculate cook loss using the formula:


$$\begin{array}{l} Cook\;loss,\;\%=\left[\left(initial\;weight,\;kgcooked\;weight,\;kg\right)÷initial\;weight,\;kg\right]\times 100 \end{array}$$

Four cores with a width of 1.25 cm were obtained parallel to muscle fibers and sheared using a Texture Analyzer.HD Plus (Texture Technologies Corp., Scarsdale, NY/Stable Microsystems, Goldalming, UK) fitted with a WBSF blade as outlined by Richardson (2018). Cores were sheared perpendicular to the muscle fiber orientation with a speed of 3.33mm/s and a load cell capacity of 100 kg. Force to shear was recorded and the average of four cores was reported.

### Statistical Analysis

Data were analyzed using the MIXED procedure of SAS (SAS Inst. In., Cary, NC) as a 2 × 3 factorial arrangement of treatments (sex × Met source) in a randomized complete block design. Pen (*N* = 60) served as the experimental unit. Fixed effects were Met source, sex, and their interaction. Block (*N* = 3) served as a random variable. Least squares means were separated using the PDIFF statement. Effects were considered significant at *P *≤ 0.05. BUN data were analyzed using the MIXED procedure with repeated measures modeling (Met source × sex × phase) of treatments in a completely randomized block design. Block served as a random variable in the MIXED procedure. Least squares means were separated using the PDIFF statement. Significance was considered at *P *≤ 0.05 and trends were considered at *P* ≤ 0.10.

## Results

### Growth Performance

Initial body weight (BW; 25.30 ± 3.5 kg) did not differ (*P* ≥ 0.37) between Met sources (Table 3). However, the initial BW of barrows was greater than gilts (82.4 vs. 78.6 kg; *P* < 0.01) at trial initiation. There were no differences between Met source for ADG, ADFI, or G:F (*P *≥ 0.39) during phase 1 (days 1 to 55) when all pigs were fed a common diet. There were no differences in BW of treatment groups at day 55 (*P *= 0.43), prior to beginning treatment diets. Diets with different sources of Met were fed in the final two phases of finishing (days 56 to 83 and days 84 to 104). There were no differences between Met sources for ADG, ADFI, G:F in either phase (*P *≥ 0.25) by adding the same amount of bioavailable Met, i.e., using 65% bioefficacy for MHA relative to L-Met or DL-Met. Final BW did not differ (*P *≥ 0.45) between Met sources. Therefore, Met source did not alter (*P *≥ 0.20) overall (days 1 to 104) growth performance. As expected, there were multiple differences between sexes for growth performance. Overall, barrows were heavier than gilts with greater ADG (*P < *0.001) and overall ADFI (*P < *0.001), while gilts had greater G:F (*P < *0.02) than barrows. However, there were no interactions (*P* ≥ 0.08) between Met source and sex for any growth performance measurements.

**Table 3.** Effects of methionine source and sex on growth performance of growing-finishing pigs^*^

	Diet			Sex			*P*-values		
Item	L-Met	DL-Met	MHA	Barrows	Gilts	SEM	Diet	Sex	Diet × Sex
Pens, *n*	20	20	20	30	30				
*Phase 1 (days 0 to 27)* ^†^									
BW day 0, kg	25.6	25.7	25.7	25.3	25.3	1.84	0.99	0.37	0.99
ADG, kg/d	0.90	0.91	0.89	0.93	0.86	0.02	0.64	<0.01	0.90
ADFI, kg/d	1.85	1.85	1.81	1.88	1.79	0.05	0.72	0.08	0.62
G:F	0.489	0.491	0.492	0.50	0.48	0.01	0.96	0.08	0.20
BW day 27, kg	50.0	50.2	49.6	50.5	49.4	2.04	0.90	0.33	0.97
*Phase 2 (days 27 to 55)*									
ADG, kg/d	1.09	1.09	1.06	1.14	1.03	0.02	0.39	<0.01	0.27
ADFI, kg/d	2.67	2.69	2.65	2.88	2.46	0.07	0.75	<0.01	0.65
G:F	0.412	0.408	0.404	0.396	0.419	0.01	0.64	<0.01	0.30
BW day 55, kg	80.6	81.6	79.4	82.4	78.6	2.32	0.43	0.01	0.91
*Phase 3 (days 56 to 83)*									
ADG, kg/d	1.20	1.20	1.19	1.25	1.14	1.14	0.93	<0.01	0.26
ADFI, kg/d	3.30	3.31	3.24	3.52	3.05	0.07	0.55	<0.01	0.87
G:F	0.364	0.365	0.368	0.357	0.374	0.01	0.92	0.02	0.33
BW day 83, kg	114.0	115.2	112.6	117.4	110.5	2.12	0.38	<0.01	0.93
*Phase 4 (days 84 to 104)*									
ADG, kg/d	1.02	1.02	1.01	1.02	1.01	0.04	0.95	0.73	0.48
ADFI, kg/d	3.49	3.54	3.45	3.66	3.33	0.11	0.25	<0.01	0.54
G:F	0.294	0.289	0.296	0.280	0.306	0.01	0.75	<0.01	0.23
BW day 104, kg	135.5	136.6	134.2	139.1	131.8	2.40	0.45	<0.01	0.89
*Overall (days 0 to 104)*									
ADG, kg/d	1.06	1.05	1.04	1.05	1.01	0.02	0.55	<0.01	0.79
ADFI, kg/d	2.80	2.84	2.76	2.95	2.64	0.07	0.20	<0.01	0.69
G:F	0.379	0.372	0.379	0.371	0.382	0.01	0.35	0.01	0.32

^*^Experimental dietary treatments were fed from days 56 to 104. Corn–sorghum-SBM diets where 25% of the Met + Cys requirement was met using one of the three Met sources; L-Met, DL-Met, and MHA. Abbreviations: BW, body weight, ADG, average daily gain, ADFI, average daily feed intake, G:F, Gain:Feed, MHA, Calcium salt of Met hydroxyl analog; SEM, standard error of the means

^†^Pigs were approximately 25 kg when placed on trial.

### Blood Urea Nitrogen

**Figure 1.**
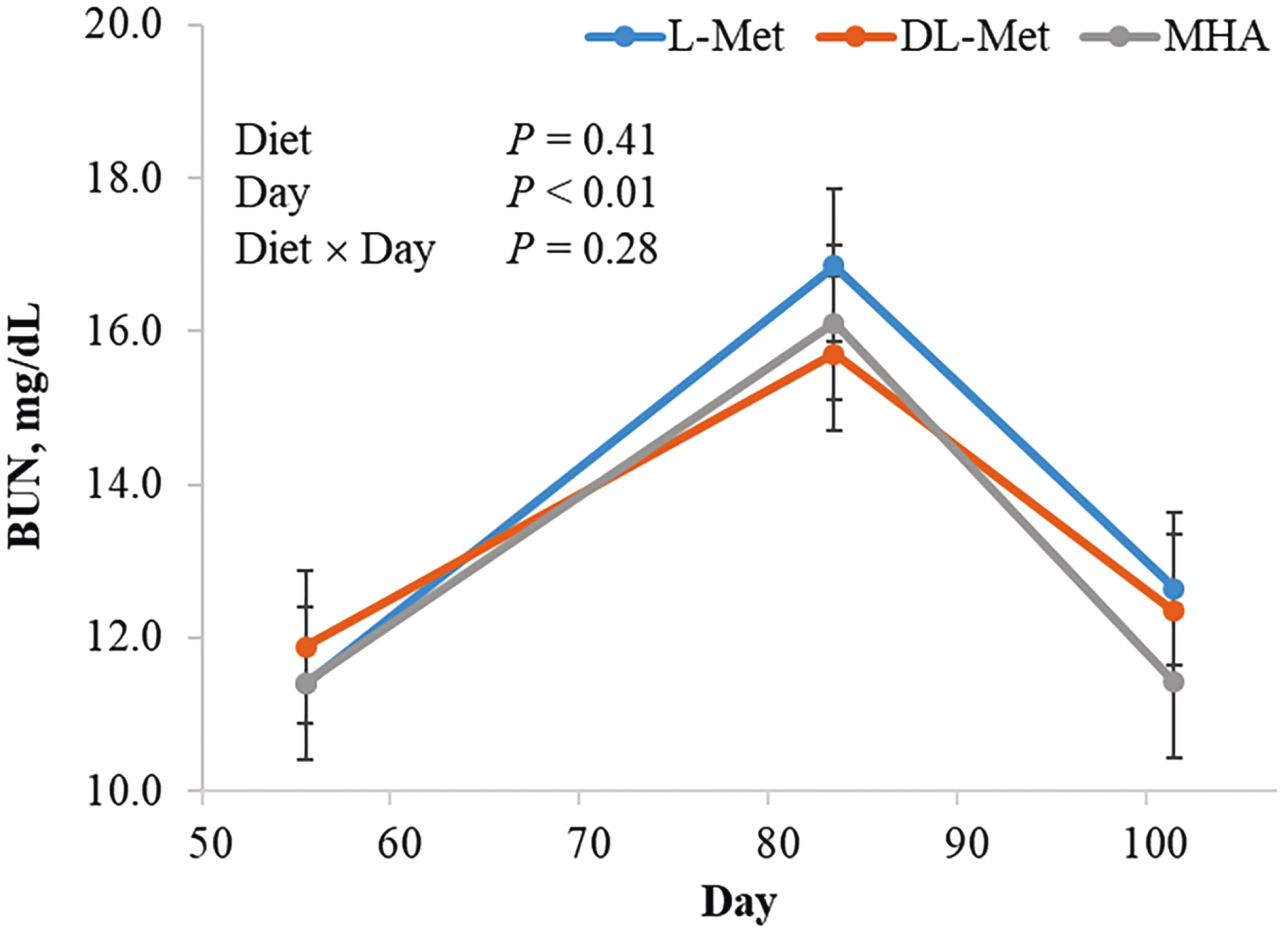
Effects of methionine source on blood urea nitrogen concentrations (mg/dL). Abbreviations: BUN, blood urea nitrogen; MHA, Calcium salt of Met hydroxyl analog.

There were no differences among Met source treatment (*P* ≥ 0.20) for BUN concentrations at any measurement time (Figure 1). On day 55, BUN did not differ (*P* = 0.67) between sexes. However, by day 83, differences in BUN emerged between sexes. Barrow BUN levels were 2.01 mg/dL greater (*P* ≤ 0.001) at phase 4 and 1.63 mg/dL greater (*P* ≥ 0.001) at d 101 compared to gilts. Phase was different from each other (*P* ≤ 0.001) with day 55 (11.55 mg/dL) and day 101 (12.12 mg/dL) being lower than day 83 (16.21 mg/dL). No treatment × sex interaction for BUN was observed (*P* ≥ 0.39).

### Carcass Characteristics

Ending live weight, HCW, and carcass yield did not differ (*P* ≥ 0.34) between Met sources (Table 4). Despite a lack of statistical significance, HCW of L-Met-fed pigs was 2.5 kg greater than MHA-fed pigs. Similarly, carcasses from DL-Met-fed pigs (103.0 kg) were also numerically heavier than MHA-fed pigs (102.0 kg), although not statistically different. Nonetheless, no differences in loin muscle area, 10th rib back-fat depth, and FFL were observed (*P* ≥ 0.51) between Met sources. Carcass differences between barrows and gilts were as expected, with barrows having greater ending live weight, HCW, and back fat thickness (*P* < 0.01) and gilts having greater standardized FFL (*P* < 0.001). No interactions between Met source and sex were observed for any carcass characteristic (*P* ≥ 0.45).

**Table 4.** Effects of methionine source and sex on carcass characteristics of pigs slaughtered at the University of Illinois Meat Science Laboratory

	Diet			Sex			*P*-values		
Item	L-Met	DL-Met	MHA	Barrows	Gilts	SEM	Diet	Sex	Diet × Sex
Pigs, *n*	20	20	20	30	30				
Ending live weight, kg	133.5	132.0	131.0	135.5	129.0	3.82	0.47	<0.01	0.79
HCW^*^, kg	104.5	103.0	102.0	106.0	100.0	2.30	0.34	<0.01	0.63
Carcass yield, %	78.35	77.89	77.93	77.93	77.82	0.65	0.39	0.12	0.45
Loin muscle area, cm^*^	52.65	52.49	52.81	53.01	53.01	1.62	0.98	0.60	0.82
10th rib back fat depth, cm	2.01	1.98	2.13	2.37	1.71	0.10	0.51	<0.01	0.81
Standardized fat-free lean^†^, %	58.58	58.77	57.90	56.04	60.80	0.85	0.75	<0.01	0.82

Abbreviations: HCW, hot carcass weight; MHA, calcium salt of met hydroxyl analog.

^*^HCW includes leaf fat.

^†^Standardized fat-free lean = ((8.588 + (0.465 × HCW, lb)−(21.896 × fat depth, in) + (3.005 × LTL area, in^2^)) ÷ HCW) × 100, (Burson and Berg, 2001).

### Carcass Cutability

Source of Met did not alter (*P* > 0.12) whole or trimmed primal absolute or relative weights, with the exception of percent bone-in picnic (*P* = 0.04; Table 5). Trimmed ham weight expressed as a percentage of chilled side weight tended to be greater in DL-Met-fed pigs (20.74%) than L-Met (20.15%) and MHA (20.44%) fed pigs. However, Met source did not alter absolute or relative weights for any ham components (Table 6). Shoulder primal cuts were not different in Met source from each other with exception of a trend in jowl weight (Table 7). Pigs fed L-Met had 0.10 kg heavier jowls than pigs fed DL-Met and MHA. On an absolute weight basis, tenderloin weights were greater for pigs fed L-Met than other Met sources (Table 8). However, differences in absolute weight did translate into a tendency (*P* = 0.08) in tenderloin as a percent of chilled side weight. Given a lack of differences in primal cut weights, Met source had no effect on bone-in carcass-cutting yield, bone-in loin-cutting yield, or boneless carcass-cutting yield (Table 9).

**Table 5.** Effects of methionine source and sex on whole and trimmed primal cuts

	Diet			Sex			*P*-values		
Item	L-Met	DL-Met	MHA	Barrows	Gilts	SEM	Diet	Sex	Diet × Sex
Pigs, *n*	20	20	20	30	30				
Chilled side wt^*^, kg	48.95	48.41	47.64	49.56	47.10	1.15	0.34	<0.01	0.78
Whole shoulder, kg	10.38	10.44	10.08	10.65	9.94	0.24	0.27	<0.01	0.58
% chilled side wt	21.21	21.56	21.17	21.50	21.13	0.18	0.20	0.06	0.50
Bone-in Boston, kg	4.17	4.14	3.98	4.23	3.95	0.17	0.21	<0.01	0.66
% chilled side wt	8.50	8.53	8.34	8.54	8.37	0.21	0.44	0.22	0.15
Bone-in picnic, kg	5.45	5.54	5.34	5.59	5.30	0.10	0.33	0.01	0.09
% chilled side wt	11.17	11.48	11.24	11.30	11.30	0.18	0.22	0.99	0.04
Whole loin, kg	14.39	14.02	13.93	14.60	13.64	0.38	0.32	<0.01	0.87
% chilled side wt	29.38	28.94	29.26	29.44	28.95	0.32	0.41	0.08	0.99
Trimmed loin, kg	11.63	11.45	11.30	11.56	11.36	0.25	0.39	0.30	0.93
% chilled side wt	23.81	23.69	23.75	23.34	24.15	0.24	0.91	<0.01	0.66
Whole ham, kg	11.57	11.71	11.42	11.68	11.45	0.16	0.45	0.23	0.40
% chilled side wt	23.70	24.22	23.97	23.58	24.35	0.44	0.14	<0.01	0.18
Trimmed ham, kg	9.85	10.04	9.75	9.92	9.84	0.16	0.39	0.66	0.43
% chilled side wt	20.15	20.74	20.44	20.01	20.87	0.53	0.08	<0.01	0.18
Natural fall belly, kg	7.86	7.52	7.58	7.91	7.40	0.39	0.13	<0.01	0.77
% chilled side wt	16.03	15.53	15.89	15.94	15.69	0.56	0.13	0.25	0.42
Spareribs, kg	1.74	1.77	1.68	1.76	1.70	0.04	0.12	0.08	0.70
% chilled side wt	3.55	3.67	3.54	3.55	3.62	0.05	0.15	0.23	0.56
*Miscellaneous cuts*									
Standardized trim, kg	0.27	0.25	0.26	0.29	0.23	0.05	0.42	<0.01	0.70
Leaf fat, kg	0.89	0.83	0.89	0.95	0.79	0.05	0.53	<0.01	0.37
Front and back foot, kg	1.08	1.08	1.05	1.07	1.07	0.03	0.03	0.95	0.65

^*^Chilled side weight is on the left side and excludes leaf fat and standardized trim.

Abbreviations: MHA, calcium salt of met hydroxyl analogue.

**Table 6.** Effects of methionine source and sex on ham primal cuts

	Diet			Sex			*P*-values		
Item	L-Met	DL-Met	MHA	Barrows	Gilts	SEM	Diet	Sex	Diet × Sex
Pigs, *n*	20	20	20	30	30				
Inside ham, kg	1.89	1.92	1.83	0.03	1.89	0.04	0.23	0.66	0.31
% chilled side wt	3.89	3.99	3.87	3.79	4.04	0.07	0.37	<0.01	0.23
Outside ham, kg	2.51	2.55	2.51	2.48	2.54	0.05	0.43	0.25	0.38
% chilled side wt	5.17	5.30	5.19	5.01	5.43	0.08	0.41	<0.01	0.17
Knuckle, kg	1.42	1.44	1.39	1.40	1.44	0.03	0.49	0.18	0.16
% chilled side wt	2.91	2.97	2.92	2.82	3.05	3.05	0.64	<0.01	0.09
Inner shank, kg	0.69	0.70	0.68	0.70	0.68	0.02	0.78	0.25	0.59
% chilled side wt	1.43	1.45	1.44	1.42	1.46	0.03	0.78	0.22	0.47
Lite butt, kg	0.24	0.25	0.26	0.24	0.26	0.04	0.53	0.27	0.34
% chilled side wt	0.49	0.53	0.54	0.49	0.55	0.09	0.31	0.04	0.42
Boneless ham^*^, kg	5.82	5.91	5.69	5.75	5.87	0.10	0.28	0.28	0.22
% chilled side wt	11.97	12.25	11.97	11.61	12.52	0.20	0.29	<0.01	0.07

^*^Boneless ham = inside ham (NAMP #402F), kg + outside ham (NAMP #402E), kg + knuckle (NAMP #402H), kg.

Abbreviations: MHA, calcium salt of met hydroxyl analogue.

**Table 7.** Effects of methionine source and sex on shoulder primal cuts

	Diet			Sex			*P*-values		
Item	L-Met	DL-Met	MHA	Barrows	Gilts	SEM	Diet	Sex	Diet × Sex
Pens, *n*	20	20	20	30	30				
Boneless Boston, kg	3.85	3.83	3.69	3.92	3.66	0.14	0.26	<0.01	0.68
% chilled side wt	7.86	7.90	7.74	7.91	7.76	0.18	0.52	0.22	0.16
Boneless picnic, kg	4.03	4.06	3.93	4.13	3.88	0.08	0.49	0.01	0.23
% chilled side wt	8.26	8.39	8.27	8.35	8.27	0.14	0.64	0.54	0.16
Neck bones, kg	0.83	0.80	0.83	0.82	0.82	0.05	0.75	0.88	0.90
% chilled side wt	1.68	1.66	1.75	1.65	1.74	0.08	0.51	0.19	0.83
Jowl, kg	1.43	1.33	1.33	1.41	1.33	0.04	0.08	0.05	0.78
% chilled side wt	2.93	2.76	2.81	2.84	2.82	0.11	0.13	0.80	0.90
Clear plate, kg	0.75	0.74	0.75	0.82	0.68	0.03	0.93	<0.01	0.74
% chilled side wt	1.53	1.53	1.58	1.65	1.45	0.06	0.67	<0.01	0.82
Boneless shoulder^*^, kg	7.89	7.89	7.89	8.06	7.54	0.19	0.27	<0.01	0.79
% chilled side wt	16.12	16.29	16.00	16.25	16.02	0.16	0.33	0.14	0.70

^*^Boneless shoulder = boneless Boston butt (NAMP # 406A), kg + boneless picnic (NAMP #405A), kg.

Abbreviations: MHA, calcium salt of met hydroxyl analogue.

**Table 8.** Effects of methionine source and sex on loin primal cuts

	Diet			Sex			*P*-values		
Item	L-Met	DL-Met	MHA	Barrows	Gilts	SEM	Diet	Sex	Diet × Sex
Pigs, *n*	20	20	20	30	30				
Canadian Back, kg	3.75	3.68	3.60	3.65	3.70	0.08	0.38	0.51	0.54
% chilled side wt	7.69	7.64	7.59	7.38	7.90	0.12	0.84	<0.01	0.67
Tenderloin, kg	0.55ª	0.52^b^	0.52^b^	0.53	0.53	0.02	0.05	0.62	0.26
% chilled side wt	1.14	1.08	1.08	1.08	1.12	0.05	0.08	0.04	0.16
Sirloin, kg	1.00	0.99	0.95	0.95	1.01	0.05	0.48	0.07	0.32
% chilled side wt	2.04	2.04	2.00	1.92	2.13	0.09	0.73	<0.01	0.09
Backribs, kg	0.85	0.87	0.84	0.90	0.81	0.02	0.65	<0.01	0.44
% chilled side wt	1.74	1.79	1.76	1.81	1.71	0.07	0.61	0.05	0.47
Backbone, kg	2.23	2.17	2.19	2.17	2.23	0.14	0.72	0.29	0.22
% chilled side wt	4.54	4.48	4.60	4.37	4.71	0.19	0.64	<0.01	0.10
Boneless loin^*^, kg	5.30	5.20	5.07	5.14	5.24	0.14	0.26	0.33	0.79
% chilled side wt	10.86	10.76	10.66	10.37	11.15	0.19	0.65	<0.01	0.77

^*^Boneless loin = Canadian back loin (NAMP #414), kg + tenderloin (NAMP #415A), kg + sirloin (NAMP #413D), kg.

Abbreviations: MHA, calcium salt of met hydroxyl analogue.

**Table 9.** Effects of methionine source and sex on carcass cutability

	Diet			Sex			*P*-values		
Item	L-Met	DL-Met	MHA	Barrows	Gilts	SEM	Diet	Sex	Diet × Sex
Pigs, *n*	20	20	20	30	30				
Bone-in carcass cutting yield^*^, %	79.65	79.95	79.65	79.13	80.38	0.66	0.66	<0.01	0.65
Bone-in lean cutting yield^†^, %	63.63	64.44	63.77	63.77	64.70	0.67	0.20	<0.01	0.59
Boneless carcass cutting yield^‡^, %	54.97	54.82	54.52	54.17	55.36	0.73	0.56	<0.01	0.66

^*^Bone-in carcass-cutting yield = [(trimmed ham, kg + bone-in Boston, kg + bone-in picnic, kg + trimmed loin, kg + natural fall belly, kg) ÷ left side chilled weight, kg] × 100.

^†^Bone-in lean cutting yield = [(trimmed ham, kg + bone-in Boston, kg + bone-in picnic, kg + trimmed loin, kg) ÷ left side chilled weight, kg] × 100.

^‡^Boneless carcass-cutting yield = [(inside ham, kg + outside ham, kg + knuckle, kg) + (Canadian back loin, kg + tenderloin, kg + sirloin, kg) + (boneless Boston, kg + boneless picnic, kg) + (belly, kg)) ÷ left side chilled weight] × 100.

Abbreviations: MHA, calcium salt of met hydroxyl analogue.

Generally, barrow carcasses produced heavier whole primal cuts on an absolute basis but no differences as a proportion of chilled side weight. For most trimmed primals, no differences in absolute weight were detected between sexes, but trimmed primals comprised a greater percentage of chilled side weight for gilts than barrows. No meaningful interactions between treatment and sex for any primal cut.

### Loin and Chop Quality

There were no differences between Met sources (Table 10) for 45 min pH (*P* = 0.67) or ultimate pH (*P* = 0.24). Ventral loin visual color, marbling, and subjective firmness were not different (*P* ≥ 0.41) between Met sources. While ventral loin L*, a*, and b* did not differ between Met sources (*P* ≥ 0.25), there was a tendency for an interaction (*P* < 0.07) between Met source × sex for ventral loin surface redness. Barrows fed DL-Met or MHA and gilts fed L-Met tended to have redder loins (a*) than barrows fed L-Met and gilts fed DL-Met or MHA.

**Table 10.** Effects of methionine source and sex on early loin and chop face quality and color^*^

	Diet			Sex			*P*-values		
Item	L-Met	DL-Met	MHA	Barrows	Gilts	SEM	Diet	Sex	Diet × Sex
Pens, *n*	20	20	20	30	30				
*Loin*									
Visual color^†^	3.08	3.25	3.08	3.13	3.13	0.11	0.41	0.99	0.14
Visual marbling^‡^	1.58	1.60	1.58	1.70	1.47	0.10	0.98	0.05	0.53
Subjective firmness^‖^	3.09	3.14	3.14	3.12	3.12	0.13	0.93	0.95	0.80
Lightness^$^, L*	49.03	48.63	50.51	50.19	48.59	0.82	0.25	0.10	0.91
Redness^¶^, a*	9.40	9.97	10.35	10.32	9.50	0.77	0.25	0.08	0.06
Yellowness^**^, b*	5.48	5.50	5.91	6.05	5.21	1.31	0.61	0.04	0.17
45min 10th rib pH	6.06	6.12	6.16	6.18	6.05	0.07	0.67	0.12	0.22
Loin pH	5.45	5.48	5.45	5.49	5.44	0.04	0.24	<0.01	0.26
Drip loss^††^, %	7.03	5.58	6.68	6.45	6.41	6.41	0.09	0.95	0.72
*Chop*									
Visual color	2.99	3.29	3.14	3.22	3.07	0.15	0.10	0.18	0.91
Visual marbling	1.67	1.82	1.62	1.92	1.49	0.17	0.52	<0.01	0.63
Subjective firmness	2.70	2.95	2.85	2.97	2.70	0.13	0.38	0.08	0.21
Lightness, L*	52.89	52.34	54.30	53.35	53.00	1.99	0.36	0.77	0.27
Redness, a*	8.59	8.63	9.64	9.23	8.68	1.39	0.19	0.30	0.47
Yellowness, b*	5.70	5.40	6.58	6.19	5.60	1.67	0.18	0.28	0.31
Moisture, %	73.86	73.94	73.79	73.59	74.14	0.21	0.84	0.01	0.89
Extractable Lipid, %	2.44	2.41	2.39	2.75	2.08	0.15	0.97	<0.01	0.81
Warner-Bratzler shear force^‡‡^, kg	3.03	3.06	3.10	2.96	3.16	0.09	0.85	0.06	0.72
Cook loss^‖‖^, %	18.18	16.20	18.50	17.33	17.92	0.74	0.07	0.49	0.58

^*^Early postmortem traits were evaluated 1 d postmortem;

^†^NPPC color based on the 1999 standards measured in half point increments where 1 = palest, 6 = darkest.

^‡^NPPC marbling based on the 1999 standards measured in half point increments where 1 = least amount of marbling, 6 = greatest amount of marbling.

^‖^NPPC firmness based on the 1991 scale measured in half point increments where 1 = softest, 5 = firmest.

^$^ L* measures darkness (0) to lightness (100; greater L* indicates a lighter color).

^¶^ a* measures redness (greater a* indicates a redder color).

^**^ b* measures yellowness (greater b* indicates a more yellow color).

^††^Drip loss = [(initial weight, kg—final weight, kg) ÷ (initial weight, kg)] × 100.

^‡‡^Includes Warner-Bratzler shear force evaluated on chops cooked to 63°C.

^‖‖^Cook loss = [(initial weight, kg—cooked weight, kg) ÷ initial weight, kg] × 100.

Abbreviations: MHA, met hydroxyl analogue.

Chop visual color tended (*P *= 0.10) to be less in pigs fed L-Met (2.99) than DL-Met (3.29) and MHA (3.14). Despite the tendency to chop visual color, chop visual marbling (*P =* 0.52) and subjective firmness (*P =* 0.38) were not different between Met sources. There were no differences between Met sources for objective color measurements (*P* ≥ 0.18). Chop moisture (*P = *0.84) and extractable lipids (*P = *0.97) did not differ between Met sources. Furthermore, Met sources were not different in objective tenderness (WBSF). While minimal differences in quality were observed, there was a tendency for chops from pigs fed DL-Met to have reduced drip loss (*P = *0.09) and cook loss (*P = *0.07) in comparison to chops from L-Met and MHA-fed pigs.

Sexes did not differ in ventral loin or chop visual color (*P *≥ 0.18). Conversely, visual marbling (*P *≤ 0.01) was greater in barrows than in gilts. Chop objective color did not differ between sexes (*P *= 0.28); however, barrows had yellower ventral loin color (b*) than gilts (*P *= 0.04). For proximate composition, chops from gilts (74.14%) had 0.55 units greater (*P *≤ 0.05) moisture than barrows (73.59%), but barrows (2.75%) had 0.67 units greater (*P *≤ 0.001) extractable lipid compared to gilts (2.08%).

### Fresh Belly Quality

Fresh belly length and width dimensions did not differ (*P *≥ 0.56) with Met source (Table 11). Likewise, there were no differences in belly thickness (*P* = 0.95) or flop distance (*P* = 0.59), both indicators of belly quality. There were no interactions between Met source and sex for any belly quality trait (*P* ≥ 0.75). As expected, barrow carcasses produced thicker bellies (*P ≤ *0.01) with greater (*P *≤ 0.01) flop distances than ones from gilts.

**Table 11.** Effects of methionine source and sex on fresh belly characteristics

	Diet			Sex			*P*-values		
Item	L-Met	DL-Met	MHA	Barrows	Gilts	SEM	Diet	Sex	Diet × Sex
Pens, n	20	20	20	30	30				
Length	71.33	70.74	70.58	71.14	70.64	1.45	0.56	0.41	0.75
Width	29.44	29.13	29.19	29.33	29.18	0.89	0.87	0.78	0.98
Thickness^*^, cm	3.65	3.62	3.65	3.85	3.43	0.12	0.95	<0.01	0.77
Flop, cm	29.74	28.00	30.39	32.06	26.69	2.08	0.59	<0.01	0.99

^*^Thickness was an average of measurements from 8 locations from the anterior to posterior.

Abbreviations: MHA, calcium salt of met hydroxyl analogue.

## Discussion

Methionine needs to be supplemented to meet the needs of pigs. Although L-Met is available for the swine industry, DL-Met and MHA are still the two common forms used in swine diets. When differences in bioefficacy were corrected for MHA, no differences in growth performance were observed in pigs and broilers fed diets supplemented with DL-Met or MHA (Lemme et al., 2002; Dilger & Baker, 2008; Zelenka et al., 2013; Albrecht et al. 2019; Pokoo-Aikins et al. 2022). In the present study, when different Met sources were used to supply 25% of SID Met + Cys requirement based on 65% bioefficacy for MHA, growth performance did not differ. Similarly, carcass composition and cutability were not different across treatments. Dietary Met sources did not affect HCW, loin muscle area, and 10th rib back fat. This mirrors the findings of Yuan et al. (2023) who reported no effect of Met source on carcass characteristics. Presently, no other studies have evaluated the effect of Met source on carcass cutability or primal yields. Findings of the current study would suggest that while primal weights may exhibit slight numerical differences, no statistically meaningful differences were observed that would prove insight that one Met source is superior to another. Minimal differences in carcass composition and cutability in this trial provide information to pork producers that the choice of Met source should not influence cutout values.

Previous reports in both pigs and chicken suggest improvements in meat quality with increasing dietary Met concentrations, however, reports of the effects of Met source on meat quality traits are mixed. Albrecht et al. (2019) reported increasing methionine concentrations reduced chicken breast lightness (L*). In pigs, increasing Met concentrations to five times the requirement during the finishing phase (70 to 105 kg) increased loin pH and WHC (Lebret et al., 2018). However, when supplemented at the same concentrations, Albrecht et al. (2019) reported no effect of Met source on breast pH, drip loss, cook loss, or color traits. In contrast, Drażbo et al. (2015) reported methionine source did impact breast pH, with MHA demonstrating increased pH over breasts from birds fed DL-Met. However, pH differences reported were minimal representing a 0.1 unit change in ultimate pH (Drażbo et al., 2015).

Yuan et al. (2023) reported an interaction between Met source and sex for loin chop pH at 45 min postmortem, with gilts fed MHA exhibiting increased pH. While it was hypothesized that this difference may be the result of changes in metabolism evidenced by greater protein kinase AMP-activated catalytic subunit alpha 2, no differences in loin pH were observed by 24 h postmortem. In the present study, no differences in loin pH were observed at either 45 min or 24 h postmortem. Tendencies for differences in chop visual color were observed between Met source, but all differences were less than 0.5 units (the smallest increment of scoring using the NPPC color scoring scale; Burson & Berg, 2001). While a tendency for an interaction between Met source and sex was observed for ventral loin redness (a*), numerical differences between sex and Met source combinations indicate one Met source is not superior to another as all values differed by less than 1 a* unit. Differences of this magnitude are not generally considered to translate into visually detectable differences in redness by consumers (Zhu & Brewer, 1999).

A tendency for improved water holding capacity from reduced drip loss and cook loss was observed for DL-Met compared to L-Met and MHA treatments. While water holding capacity has an important role in post-processing yields and sensory juiciness, it is unlikely the approximately 1.5-unit improvement in drip loss and 2-unit improvement in cook loss would translate into a meaningful difference in consumer palatability. Sensory juiciness has been reported to account for less than 8% of consumers’ perceptions of overall palatability (O’Quinn et al., 2018) and no differences in extractable moisture were observed. In another study where DL-Met and MHA-FA diets contained 125% of SID sulfur amino acid, no differences in objective tenderness (WBSF) were observed when chops were cooked to 70 °C (Yuan et al., 2023). As sensory tenderness has been reported to comprise approximately 43% of consumers’ perceptions of overall palatability (O’Quinn et al., 2018), the present lack of differences in objective tenderness, as measured through shear force, indicates Met source would not affect the loin chop eating experience.

Different than beef, as the majority of pork products purchased by US consumers are further processed (Pork Checkoff, 2015) mitigating negative effects on belly quality is important to the production of bacon. Met source did not alter belly thickness or flop distance suggesting that no differences in bacon processing would result from different Met sources. This suggests that Met source would also not impact fatty acid composition or iodine values.

## Conclusion

In conclusion, methionine sources had little effect on carcass characteristics and meat quality of finishing pigs when using an average bioefficacy of 65% for MHA relative to L-Met or DL-Met. While methionine sources may be processed differently within the body, utilization of different sources did not impact growth performance, carcass characteristics, or meat quality in pigs. The lack of differences indicates that when used similarly to the present study, there may be little advantage or disadvantage for carcass yield and quality to using one Met source over another.

## Supplementary Material

txae088_suppl_Supplementary_Table_S1
