# Supplementary material for: Effects of supplemental methionine sources in finishing pig diets on growth performance, carcass characteristics, cutting yields, and meat quality
Source: Transl Anim Sci. 2024 May 27;8:txae088. doi: 10.1093/tas/txae088 (PMC11165637; doi:10.1093/tas/txae088)
Supplement: txae088_suppl_Supplementary_Table_S1 [file txae088_suppl_supplementary_table_s1.pdf]

Supplemental Table 1. Ingredient and nutrient composition of diets for common Phases 1 and 2[1]

Item	Phase 1 (d 0 - 27)	Phase 2 (d 28 - 55)
Ingredient, %		
Ground corn	56.58	64.64
Sorghum	10.00	10.00
Soybean meal	30.00	22.00
Soybean oil	1.00	1.00
Limestone	0.83	0.79
Dicalcium phosphate	0.92	0.80
L-Lysine.HCl	0.09	0.17
L-Threonine	0.03	0.05
Salt	0.40	0.40
Vitamin-mineral premix[2]	0.15	0.15
Calculated values		
ME, kcal/kg	3,346	3,356
Crude protein, %	19.9	16.8
Ca, %	0.66	0.59
P[3], %	0.31	0.27
Total AA[4], %		
Arg	1.18	0.95
His	0.48	0.40
Ile	0.73	0.60
Leu	1.53	1.34
Lys	0.98	0.85
Met	0.28	0.24
Met + Cys	0.55	0.48
Phe	0.86	0.72
Thr	0.65	0.56
Trp	0.22	0.17
Val	0.79	0.66

[1]Diets for Phases 1 and 2 are formulated to meet nutrient requirements for 25-50 and 50-75 kg pigs, respectively.

[2]The vitamin-mineral premix will provide the following quantities of vitamins and micro-minerals per kilogram of complete diet: Vitamin A as retinyl acetate, 11,150 IU; vitamin D3 as cholecalciferol, 2,210 IU; vitamin E as DL-alpha tocopheryl acetate, 66 IU; vitamin K as menadione nicotinamide bisulfate, 1.42 mg; thiamin as thiamine mononitrate, 1.10 mg; riboflavin, 6.59 mg; pyridoxine as pyridoxine hydrochloride, 1.00 mg; vitamin B12, 0.03 mg; D-pantothenic acid as D-calcium pantothenate, 23.6 mg; niacin, 44.1 mg; folic acid, 1.59 mg; biotin, 0.44 mg; Cu, 20 mg as copper chloride; Fe, 125 mg as iron sulfate; I, 1.26 mg as ethylenediamine dihydriodide; Mn, 60.2 mg as manganese hydroxychloride; Se, 0.30 mg as sodium selenite and selenium yeast; and Zn, 125.1 mg as zinc hydroxychloride.

[3]Standardized total tract digestible P.

[4]Amino acids are indicated as standardized ileal digestible AA based on AMINODat 5.0 Platinum version, 2016. (Evonik Nutrition & Care GmbH, Hanau-Wolfgang, Germany)
